# Caps Captioning: A Modern Image Captioning Approach Based on Improved Capsule Network

**DOI:** 10.3390/s22218376

**Published:** 2022-11-01

**Authors:** Shima Javanmardi, Ali Mohammad Latif, Mohammad Taghi Sadeghi, Mehrdad Jahanbanifard, Marcello Bonsangue, Fons J. Verbeek

**Affiliations:** 1 Section Imaging and Bioinformatics, Leiden Institute of Advanced Computer Science (LIACS), Leiden University, Niels Bohrweg 1, 2333 CA Leiden, The Netherlands; 2 Computer Engineering Department, Yazd University, Yazd P.O. Box 8915818411, Iran; 3 Electrical Engineering Department, Yazd University, Yazd P.O. Box 89195741, Iran

**Keywords:** image captioning, deep learning, Convolution Neural Network, natural language processing

## Abstract

In image captioning models, the main challenge in describing an image is identifying all the objects by precisely considering the relationships between the objects and producing various captions. Over the past few years, many methods have been proposed, from an attribute-to-attribute comparison approach to handling issues related to semantics and their relationships. Despite the improvements, the existing techniques suffer from inadequate positional and geometrical attributes concepts. The reason is that most of the abovementioned approaches depend on Convolutional Neural Networks (CNNs) for object detection. CNN is notorious for failing to detect equivariance and rotational invariance in objects. Moreover, the pooling layers in CNNs cause valuable information to be lost. Inspired by the recent successful approaches, this paper introduces a novel framework for extracting meaningful descriptions based on a parallelized capsule network that describes the content of images through a high level of understanding of the semantic contents of an image. The main contribution of this paper is proposing a new method that not only overrides the limitations of CNNs but also generates descriptions with a wide variety of words by using Wikipedia. In our framework, capsules focus on the generation of meaningful descriptions with more detailed spatial and geometrical attributes for a given set of images by considering the position of the entities as well as their relationships. Qualitative experiments on the benchmark dataset MS-COCO show that our framework outperforms state-of-the-art image captioning models when describing the semantic content of the images.

## 1. Introduction

Automatic image captioning is a challenging problem in computer vision, and it aims to generate rich content and human-understandable descriptions for given images [1]. With the increase in the volume of digital images, we must deal with many different image resources on the Internet, i.e., news articles, advertisements, blogs, and the like. As most images have no description, their user-driven interpretation is challenging, and even when a description is present, manually checking that it corresponds to the image is time-consuming. Therefore, the increasing volume of images asks for automatic image captioning approaches to describe the content of images. Describing the content of images has many applications, such as scene understanding and image retrieval in several use cases including biomedicine, business, education, digital libraries, and web search engines [2]. For example, image captioning effectively allows blind people to comprehend and perceive their surroundings.

**Figure 1:**
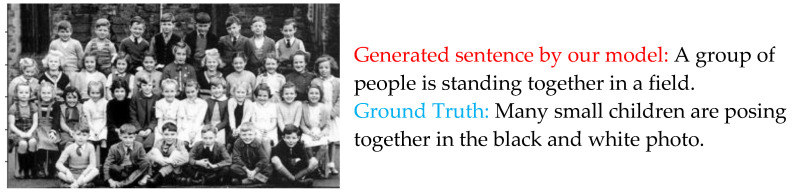
An example of an image description with the proposed model.

The performance of image captioning models is closely related to the quality of extracted features from images. The power of the language model can help to generate accurate and meaningful descriptions related to image content. Considering the semantic relationships between the identified objects within the image is essential in the image caption generation task. However, identifying the objects (i.e., the nouns in the caption) within an image is still challenging. Moreover, finding their interaction (i.e., the verbs in the caption) is extremely difficult. In fact, expressing object interaction by natural language as semantic knowledge, either as verbs or adverbial compositions, is the core issue in image captioning. Figure 1 shows an example image for which our model has generated its corresponding caption and one given manually by a human. In both captions, the relationship (standing, posing) among the objects (group of people, small children) plays an important role in understanding the picture.

The visual content of the images alone cannot always be completely interpreted. Recent image captioning methods use deep learning algorithms to control the complexity and address the above challenges of the image captioning process [3,4,5,6,7,8]. However, they struggle with generating realistic descriptions that capture all image concepts. Other image captioning models use convolutional neural networks (CNN) as an image feature extractor. These networks cannot significantly identify prominent image objects and their relationships to generate a meaningful description for the image. Additionally, CNN needs a lot of data to learn, and using pooling layers in CNN leads to valuable information loss.

In this paper, we develop a novel method that (1) overcomes the limitations of CNNs, (2) generates descriptions with a non-restricted variety of words, and (3) is capable to describe the relationships between the objects. We use a novel encoder–decoder mechanism that addresses these challenges by using a capsule network (CapsNet) [9]. The result is a set of meaningful descriptions for the image via a language model. CapsNet can effectively compensate for the shortcomings of a CNN by detecting tissue overlap characteristics [10]. In CapsNet, more salient spatial features and geometrical attributes, such as direction, size, scale, and object attributions, can be represented for each input. This aspect of CapsNet contrasts with CNN since the lack of local invariance features produces excessive variations of global discriminating outputs [11]. In addition, our model employs an external knowledge base, i.e., Wikipedia, aiming to accomplish augmented textual training data to generate more meaningful and diverse captions.

More specifically, in our model, the encoder–decoder system is employed to describe the content of images in natural language. The encoder extracts attributes from the features of the image together with semantic relationships between those attributes by a CNN and a CapsNet. The output of the encoder is three sequences of indexes. The first one declares the visual content and high-level concepts within each image. The second sequence of indexes is the corresponding textual information extracted from Wikipedia based on the predicted labels of the images, and the last sequence represents the descriptions of each image as already present in the dataset. These fixed-length attribute vectors are fed to the recurrent neural networks (RNN) as a decoder to generate a caption by a language model. The main contributions of our work are as follows:

- The development of a novel parallel structure for a capsule network can capture more comprehensive information about the objects within an image by considering their relationships.
- The use of Wikipedia as an external knowledge base for enrichment of all the textual training information and generating out-of-domain representation when describing the content of the image
- The application of our framework on the MSCOCO large-scale dataset. As mentioned in [11], using large-scale dataset including RGB images requires a huge number of resources because of the architecture of capsule networks.
- We performed a benchmarking towards a list of existing state-of-the-art models.

This paper is organized as follows: Section 2 presents an overview of the related literature and models in image captioning. All the employed models and the proposed method with the design of the framework are presented in Section 3. In Section 4 and Section 5, the reader can find the descriptions of all experiments and the study results, followed by the conclusions in Section 6.

For assessing the results, we used standard discrete natural language processing metrics such as BLEU 1–4 [12], ROUGE [13], and METEOR [14], showing a more accurate description of the input image when compared to existing state-of-the-art models.

## 2. Related Work

Image captioning is a popular research topic in computer vision and natural language processing. Generating an accurate textual explanation that describes the content of an image is accomplished by understanding the visual content of the image. Recently, the interest in image captioning has broadened with the development of benchmark datasets such as MS-COCO [15], Flickr 8K [16], and Flickr 30K [17].

Current image captioning models can be categorized into template-based, retrieval-based, and neural network-based models. The template-based models [18,19,20] first detect all the image attributes using image classification and object detection methods. These methods generate captions by filling in pre-defined templates from the identified objects. This approach produces too flexible captions that cannot correctly describe the relationships between attributes [21].

Retrieval-based models [22,23,24] create a pool of similar images in an image database and rank the retrieved images by measuring their similarities and then change the found image descriptions to create a new description for the queried image. The usefulness of this strategy is severely constrained when dealing with images that are not in the dataset and thus not classified, i.e., unseen.

The neural network-based models are inspired by the success of deep neural networks in machine learning tasks and use in an encoder–decoder architecture [25,26,27,28,29,30,31,32,33,34,35]. An encoder extracts image contents by a CNN, a module associates contents to words, and a decoder by an RNN is used for language modeling and creating image captions. Kiros et al. [27] proposed a multimodal language model that jointly learned the high-level image features and word representations. Their model can generate image captions without using any default template or structure, making the model more flexible. Nevertheless, their model could not learn latent representations of the interactions between the objects in the image. Moreover, they investigated a manual algorithm including multiple modules that cannot learn from each other during the training process.

Wu et al. [25] proposed a two-phase attribute-based model for the image captioning approach based on a CNN-LSTM framework. The CNN classifier extracts the attributes as high-level semantic concepts in their framework to generate image captions. They significantly improved in generating rich captions, but their model demonstrates the problem of equally distributing semantic concepts in whole sentences [5]. They also implemented a visual question-answering model in the captioning phase using extracted information from an external knowledge base to answer a wide range of image-based questions based on the content of images.

Mason and Charniak [28] proposed a graphic retrieval model to obtain the textual description of undescribed images based on the text descriptions of similar images with the highest rank in the dataset. The constant presence of the best matches description sentence to the query image is unrealistic. A word frequency model has been used to find a smoothed assessment of the visual content of various captions. The same challenge is found in [29], in which Devlin et al. provided the nearest neighbour method for image captioning. They make a pool of captions based on training data and describe the query image based on the nearest neighbour images. Vinyals et al. [30] used a Neural Image Caption (NIC) model to generate a plain text description by maximizing the likelihood of the target sentence given the image. In NIC, the words with the highest probability are selected from outputs to be formed as an image description.

Lebret et al. [31] investigated a CNN-based image captioning approach to infer phrases that describe the image. Then all the predicted phrases are combined using a language model to create a caption. Their proposed model is an example-based method that makes the model like a large dictionary, and accurate, relevant descriptions will not always be found in the data source. Therefore, these methods are not always fine for complex data, although they avoid critical mistakes in generating captions using a language model.

You et al. [32] proposed a combined bottom-up and top-down model which selects salient regions of an image via a bottom-up mechanism and then generates the captions by applying a top-down mechanism. A similar image captioning method has been proposed by Johnson et al. [33]. They employed a convolutional localization network to predict a set of captions across the important regions of the image and generate the label sequences using a recurrent neural network. The proposed method localizes the salient regions and generates captions for each region using a language model. Finding a relationship between all these regions is always a big challenge in these approaches.

Liu et al. [3] proposed an ontology to describe the scene construction of images. Their constructed ontology can specify the object types and the special information for the objects (e.g., location, velocity). This visual and special information can transform into meaningful project information for generating captions using integrated computer vision and linguistic models.

Various improvements are made to captioning models to make the network more inventive and effective by considering visual and semantic attention to the image. For example, in Ref. [34], Yang and Liu introduced a method called ATT-BM-SOM to increase the readability of the syntax and optimize the syntactic structure of captions. This framework operates based on the attention balance mechanism and the syntax optimization module and effectively fuses image information. Their model generates high-quality captions, compensating for the lack of image information selection and syntax readability.

Training large amounts of data give machine learning models greater predictive performance. However, training massive data by machine learning may increase the execution time of the model and it could memorize the data that causes the model to overfit. In Ref. [35], Martens and Provost demonstrated that a large amount of data could lead to lower estimation variance and hence lower error with better prediction performance. However, data quality plays an important role in the performance of the model. The hypothesis is that more data may contain useful information. To this aim, Hossain et al. [36] proposed a method that leverages a combination of real and synthetic data generated by the Generative Adversarial Network (GAN). It is an efficient alternative for the techniques requiring human-annotated images, as they are labor-intensive to generate and time-consuming.

Xian and Tian [37] employed a self-guiding model to extract textual features using the multimodal LSTM model. Their model adequately describes the images without having a perfect training dataset. It is an important issue that we have considered in the research described in this paper. Recently, Reinforcement Learning (RL) methods have been incorporated into image caption generation models. Rennie et al. [26] proposed a reinforcement model for optimizing the process of image captioning. They considered a reward parameter on the results at the test time. Yan et al. [38] proposed a hierarchical model that uses the GAN and RL algorithm to produce more accurate captions for images. They measured the consistency between the generated captions and the content of images by the RL optimization process and the discriminator in the framework of GAN. For object detection and extracting salient regions from an image, they used faster R-CNN models, and then they used CNN to extract features from the proposed regions. They achieved significant improvement over the generated captions for the images.

In Section 3, the structure of the image caption generation models and the employed networks in our experiments will be discussed in more detail.

## 3. Materials and Image Captioning Methods

**Figure 2:**
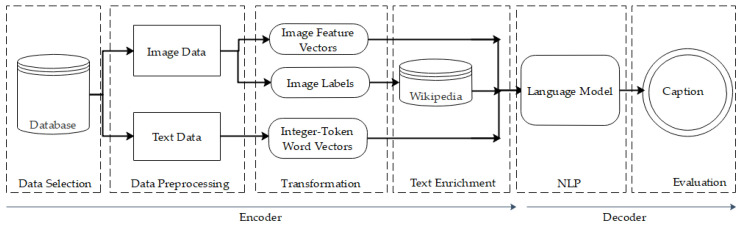
KDD methodology of the proposed model.

Following the trend of current work, as mentioned in Section 2, we use an encoder–decoder framework to create the captions of images. Understanding the image requires recognizing the objects, properties, and interactions in the encoder part. Moreover, producing sentences to describe images in the decoder requires understanding language syntax and semantics. Figure 2 illustrates the employed Knowledge Discovery Database (KDD) of our model: images and descriptions proceed separately in the data processing phase. Then in the transformation phase, all the image and text data are processed to create feature vectors for the language model. A CNN is employed for predicting the labels from the given image. In the text enrichment phase, we used Wikipedia to extract relevant information based on the predicted labels of images. Then, all the data sequences are fed to the language model in the NLP phase for tokenizing, embedding, and making word vectors from the image captions in the dataset and extracted knowledge from Wikipedia. After which, all the information is fed into the caption predictor in the evaluation section to produce a caption given the input image.

The novelty of our work consists of a new variant of the capsule network, parallelizing its basic structure to capture more comprehensive information about the objects within the image, thus leading to a more accurate description of the input image. The primary structure of the capsule network works well on a simple dataset such as MNIST, which includes images with a single object and only one channel. However, the network efficiency significantly decreases when applied to images with large special dimensions and complex datasets such as MS-COCO and Flicker. The presence of multiple channels and objects in the images increases the training time of the network and leads to weak results compared to state-of-the-art [39]. This problem happens due to inefficiency in capturing the underlying information of the image. To handle this issue, we extended the baseline network by parallelizing the convolutional layers and the primary capsules of the original CapsNet, followed by a concatenation approach to extract more complex and qualified features from the images. On the other hand, parallelizing the convolution layers reduces the dimensions of the fed features to the primary capsules and accelerates the learning process.

In the proposed image captioning model, we use CNN and CapsNet architectures to incorporate visual context from an image, which is then used as the input of a machine translation, such as an RNN architecture, to generate objective sentences in the decoder part of the framework. We applied cross-entropy loss to adjust the model weights during the sequential model training. In this section, the entire model flow is described in more detail. 

We divide the dataset into a train, validation, and test subsets. The train and validation sets are fed to the CNN and CapsNet to extract the visual features next. Transfer learning in CNN has been involved in retraining the MS-COCO dataset and extracting the visual attention of images. We have applied both the Inception-V3 or VGG16 as image feature extractors. These networks are trained on the ImageNet dataset with more than one million images of 1000 classes. Training the CapsNet is done from scratch and based on 80 categories of objects in Category Caps. Subsequently, the image features and captions are transferred to the RNN network to train the language model. 

The proposed architecture uses Inception-V3 and capsule networks to extract visual information from the images and compare all our experiments to the result of the base models. The details of these networks are shown in Table 1.

### 3.1. Inception-V3

In 2015, Google introduced GoogleNet [40]. This network reduces the computational burden of the network with a lightweight structure and has been shown to obtain better performance. The first version of the inception network includes filters of multiple sizes (1 × 1, 3 × 3, 5 × 5) to perform convolution on an input image. To reduce the network parameters and computational cost, Inception-V3 breaks down the kernels into smaller sizes (e.g., 5 × 5 kernels into two (1 × 5, 5 × 1)). This solution can extend the depth of the network and helps to prevent computation and overfitting issues. Our research demonstrated the proper performance of Inception-V3 [41]

In the encoder phase, we used the extracted features from the last fully connected layer of the Inception-V3 network and the predicted labels from the SoftMax layer.

### 3.2. VGG16

This network is one of the two networks introduced by Simonyan and Zisserman in 2014 [42]. This model has 13 convolutional layers of a 3 × 3 filter with a stride of 1 pixel followed by a max-pooling layer 2 × 2 filter of stride two and ReLU activation function. ReLU can reduce the gradient disappearance problem by providing more optimal error transmission than the sigmoid function. This network computes approximately 138 M parameters and is considered an extensive network. A pre-trained network on the ImageNet dataset extracts visual features from input images by applying the transfer learning method. VGG16 has five convolutional layers and pooling modules. These modules have respectively 64, 128, 256, 512, and 512 filters. The feature map size will be reduced in half after each module. Following [25], we considered this model a baseline because of its straightforward character. We employ the extracted features from the last fully connected layer to initialize the RNN network.

### 3.3. Capsule Network

A capsule is a set of neurons whose activity vectors indicate the posture characteristics of an entity and the length of the vector denotes the chance of that entity existing. Unlike a convolutional network, capsules save comprehensive information about the location and pose of an entity.

Hinton et al. [9] claimed that regardless of the high capability of CNNs, this network has two main disadvantages: 1—lack of rotation invariant and 2—using a pooling layer. The former causes failure in recognizing spatial relations between the objects, and the latter causes information loss due to the maximum value selection of each region. Therefore Sabour et al. [9] proposed a capsule network to address the issues mentioned above.

**Figure 3:**
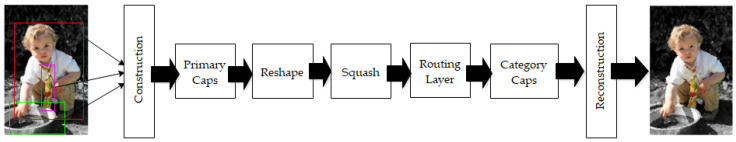
Capsule Network Architecture.

There are different concrete components in a capsule network for learning the semantic representations within the image (see Figure 3). These components map construction by reconstructing the discrepancy map from the input image. The major components of the capsule network involve the following:

- Primary capsules combine the features extracted by convolutional layers in the construction phase.
- Reshaping the extracted feature maps from the primary capsules.
- Squashing is a non-linear activation function that squashes the weighted input vector of a particular capsule. This function distributes the length of the output vector between 0 and 1.
- The dynamic routing layer produces output capsules with high agreements by automatically grouping input capsules. The pooling layers in the capsule network are replaced by a mechanism called “routing by agreement” in the rooting layer: the output of each capsule in the lower level is sent to the parent capsules in the higher level only if their features have a dependency.
- Category capsules with a marginal classification loss and a reconstruction sub-network with a reconstruction loss for recovering the original image from capsule representations.

**Figure 4:**
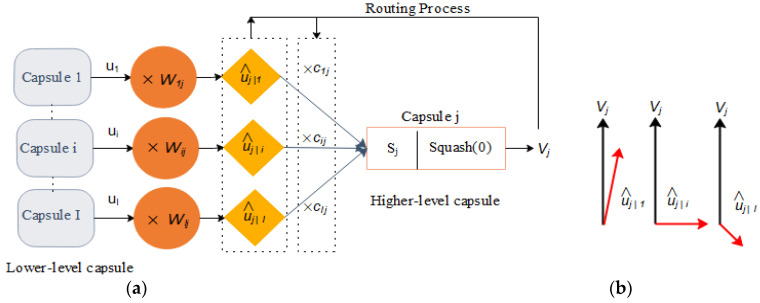
(**a**) Transferring information among capsules [1…I] and high-level capsules (**b**) routing procedure.

The operation of all these components is explained in this section in more detail. One important aspect of capsule networks is their ability to identify individual parts of objects in a single image and then represent spatial relationships between those parts. For example, in Figure 3, the CapsNet has identified three different parts of objects within the input image (tie, child, bin). The output image on the right side of the figure shows the result of the reconstruction subnetwork in the employed capsule network. Figure 4 shows the construction of a capsule and how data is routed between lower-level and higher-level capsules.

In Figure 4a, each capsule finds the appropriate parent in the next layer during the dynamic routing procedure to send its output to those capsules in the above layer. The input and output of a capsule are vectors. Given $u_{i}$ as the prediction vector of capsule i and $u_{j|i}$ as the output of parent capsule j in higher level will be computed by multiplying $u_{i}$ with a weighted matrix $w_{ij}:$

(1) $${\hat{u}}_{i|j}=w_{ij\;}.\;u_{i}$$

The length of $u_{i}$ indicates the probability of predicting a component in the image even after changing the viewing angle. The direction of $u_{i}$ represents several properties of that component, such as size and position. A weighted sum overall $\hat{u_{j|i}}$ and an intermediate coupling coefficient $c_{ij\;}$, is calculated as the total input vector to capsule j by the following function:

(2) $$s_{j}=\underset{i}{\sum }c_{ij}\hat{u_{j|i}}$$

Here, the coupling coefficient $c_{ij\;}$, are the class-specific likelihood calculated after flattening the vectors and is computed by a routing SoftMax function as follows:

(3) $$c_{ij}=\frac{\exp\left(b_{ij}\right)}{\sum_{k}\exp\left(b_{ik}\right)}$$

where $b_{ij}$ represents the log probability of connection between capsules i and j. As shown in Figure 4b, the value of $c_{ij\;}$increases when the lower-level and higher-level capsules are consistent with their predictions and decreases when they are inconsistent. Based on the original paper, this parameter is initialized at 0 in the routing by agreement procedure. Instead of applying the ReLU activation function as in VGG16 and Inception-v3, the following non-linear squashing function [9] will be calculated over the input vector in this network:

(4) $$v_{j}=\frac{{\left|\left|s_{j}\right|\right|}^{2}}{1+{\left|\left|s_{j}\right|\right|}^{2}}\frac{s_{j}\;}{\left|\left|s_{j}\right|\right|}$$

where $s_{j}$ is the input vector and $v_{j}$ is the normalized output between 0 and 1. The log probability is updated along with the routing mechanism by calculating the agreement between $v_{j}$ as the output of capsule j in the above layer and $\hat{u_{i|j}}$, as a prediction vector.

The loss function of the network for each capsule k is computed as follows:

(5) $$L_{k}=T_{k}\max{\left(0,\;l^{+}-\left|\left|V_{k}\right|\right|\right)}^{2}+\lambda \;\left(1-T_{k}\right)\;max{\left(0,\;\left|\left|V_{k}\right|\right|-l^{-}\right)}^{2}\;$$

where $L_{k}$ is loss term for one prediction, $T_{k}$ is a term equal to 1 when the class k is present; otherwise, it is 0. The upper and lower bounds of margin loss parameters, $l^{+}$ and $l^{-}$, are set to 0.9 and 0.1 [9]. It means that if an entity is present with a probability above 0.9, the loss is zero; otherwise, the loss is not zero. Regarding capsules that could not predict the correct label, if the predicted probability of all those labels is below 0.1, the margin loss is zero; otherwise, it is not zero. The parameter λ is set at 0.5 and is used for numerical stability to control the down weighting of the initial weights for the absent classes. || . || in all the equations denotes L2 norm.

### 3.4. Improved Capsule Network

In the improved version of the capsule network architecture, where we parallelized the convolution layers and primary capsules, the input image size is 229 × 229 × 3. The different architecture of the capsule network distinguishes it compared to CNN. Except for the input and output layers, the capsule network consists of primary and category capsule layers. The output of the capsules is forwarded to the decoder. The networks prevent overfitting by rebuilding the input image from the output capsules by minimizing the reconstruction loss as a regularization method in the decoder [43].

**Figure 5:**
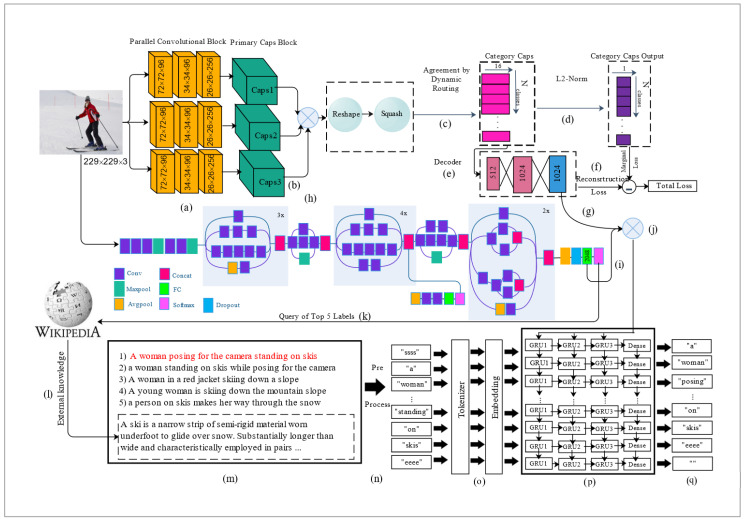
Our proposed model: a CNN and a CapsNet are applied to a given image to produce the visual features and predict the attributes of the image (**a**–**k**). The textual information of each sample comprises the descriptions of the image and the aggregated data from the external database, and a preprocessed method is applied to the text (**l**–**n**). After tokenizing and embedding process, the visual attention of the image is fed to a GRU with three levels to generate a caption to explain the content of the image (**o**–**q**).

The original capsule network has been tested on the MNIST dataset with one color channel (grayscale). However, the color of objects is an important factor in object detection and image captioning tasks. Therefore, we propose a parallelized capsule network that generates the descriptions of the images by passing the RGB images with three color channels through the three blocks of parallel convolutional layers and parallel primary capsules. The three-color channels of RGB images can store information and intuitively visualize content. Therefore, color analysis is also addressed in this parallelized structure of the capsule network, which makes the model more informative and improves the descriptiveness of image captions by extracting more qualified features from the image [44]. Adding more convolutional layers was not logical due to the increasing model complexity computational cost. The structure of the new network has been presented in Figure 5.

### 3.5. Gated Recurrent Uni

Our image captioning framework used a three-layer RNN network with a Gated Recurrent unit cell [45]. This RNN is equipped with visual features in the feature maps of CNN and CapsNet. The proposed model generates a description for each image by maximizing the probability of the current word predicted in the caption according to the following formula:

(6) $$\theta^{*}=argmax_{\theta }\underset{\left(I,M\right)}{\sum }\log\;p(M|I;\theta )$$

where θ are the parameters of the proposed model and *M* is the correct description of image *I*. Suppose $\left\{m_{0},\ldots ,\;m_{N-1}\right\}$ is a sequence of words in transcription *M* of length *N*, then log p(M|I) as the probability of generating a word for an image *I*, is as follows:

(7) $$\log\;p\left(M|I\right)=\overset{N}{\underset{t=0}{\sum }}\log\;p(m_{t}|I,m_{0},\ldots ,\;m_{N-1},c_{t}\;)$$

where t is the time step and $c_{t}$ is context vector. A two-step process feeds all the text data to the RNN network. The first step is tokenizing, and the second one is embedding. All the words in the sentences are converted into so-called integer-token vectors during tokenizing. This process is based on 10,000 most frequent and unique words in the image captions.

Throughout the embedding, all the integer-token vectors are transformed into floating-point vectors. We considered this part a decoder consisting of three GRU layers with an input size of 512. The embedding layer converts all the integer tokens into a 128-length vector. The output features initialize the GRU units from the encoder part. The governing equations in GRU are given as follows:

(8) $$r_{t}=\sigma \left(W_{r}\left[h_{t-1},x_{t}\right]+b_{r}\right)$$

(9) $$z_{t}=\sigma \left(W_{z}\left[h_{t-1},x_{t}\right]+b_{z}\right)$$

(10) $${\tilde{h}}_{t}=tan\;h\left(W_{h}\left[r_{t}ʘh_{t-1},x_{t}\right]+b_{{\tilde{h}}_{t}}\right)$$

(11) $$h_{t}=z_{t}ʘ{\tilde{h}}_{t}+\left(1-z_{t}\right)ʘh_{t-1}$$

(12) $$x_{t}=\left[E_{w}m_{t-1},c_{t}\right]$$

where $r_{t}$ is reset gate vector at instant t, $h_{t}$ is output vector of the hidden layer, ${\tilde{h}}_{t}$ is candidate activation vector, which is temporary output, $z_{t}$ is update gate vector, and $W_{r},\;W_{z},\;W_{h}$ are the weight matrices of the reset gate, the update gate, and the temporary output. All the biases corresponding to these weight matrices are represented by $b_{r},\;b_{z},\;b_{{\tilde{h}}_{t}}$. $x_{t}$ is input vector at instant t, which is based on the input embedding matrices, *E_w_*, and the one-hot encoder of the previous word, $m_{t-1}$. $c_{t}$ is the context vector extracted by the feature maps of CNN and CapsNet. The concatenation operator is applied on $E_{w}m_{t-1}$ and $c_{t}$ to make the input of the RNN network. ʘ is an element-wise product.

Eventually, we minimize the following standard cross-entropy loss function for the proposed captioning model with parameter θ and given a target ground truth $m_{1:t}^{*}$.

(13) $$L_{c}\theta =-\overset{T}{\underset{t=1}{\sum }}\log\;(p_{\theta }(m_{t}^{*}|m_{1:t-1}^{*}))$$

The performance of the implementation by different metrics is discussed in the section on evaluations and results.

### 3.6. External Knowledge

Many pipeline approaches have been proposed for image captioning by integrating knowledge in text script form. In this paper, the generated caption of an input image is obtained using “beam search”, i.e., in each iteration for training one image, we considered the top five attributes as a candidate for a query in a knowledge database to retrieve sentences. After extracting the visual features of each image using CNN and CapsNet, those five predicted attributes are used as queries to extract contextual information from the Wikipedia database for every image in the training dataset. We only selected the first three sentences for every attribute from all information retrieved from Wikipedia. Then by applying the automatic summarization method, we extract the first three sentences of retrieved text for each top five predicted label from CNN. By using this external knowledge, we enrich the descriptive information of each image. We then passed this information and all five available captions in the training set to the RNN network for generating a descriptive caption from the image.

### 3.7. Framework

The final model follows the encoder–decoder framework. The entire architecture of our proposed model is shown in Figure 5. There are three primary phases in this model. The first phase includes extracting features from the images using two deep neural networks. In this step, CapsNet and inception-V3 are used for extracting visual content from the input image concurrently. In CapsNet, at first, three parallel levels with three convolutional layers in 72 × 72 × 96, 34 × 34 × 96, and 26 × 26 × 256 sizes are applied to each channel of the image (Figure 5a). As stated in Section 3, a primary capsule block is followed by a reshaping and squashing process to take the concatenated features recognized by the convolutional and primary capsule layers and combine them to produce new features (Figure 5b). Then, the “routing by agreement” mechanism is performed rather than a pooling operation (Figure 5c). Based on this mechanism, the output of each capsule in the lower level is sent to those parent capsules in the higher level with dependency on their features. The next layer is category capsules, which indicate the membership probability of the input image in each category. The actual label masks the output of the categorical capsule layer by using the L2-norm to calculate the loss (Figure 5d). The last part of the capsule network is the decoder, which is used as a regularizer with two fully connected layers with sizes 512 and 1024 (Figure 5e).

Capsules are forced to learn features that can be used to reconstruct the input image by the decoder based on the calculated reconstruction loss. The output of the second fully connected layer is used as the image visual features vector (Figure 5f). At the same time, Inception-V3, as the second feature extractor, produces the features vector from the input image (Figure 5h). A pre-trained convolutional network is used in this step to handle the overfitting issue and increase the training time. Then, both visual feature vectors are concatenated to feed the language model (Figure 5g,i,j). All of these operations are done in the first phase.

In the second phase, in addition to five captions for each image in the dataset, we extract external knowledge from Wikipedia based on the top five labels of each image extracted from the CNN network (Figure 5k). We use the first three sentences of the description retrieved by Wikipedia (Figure 5l) for each label. Finally, the information from the first two phases is fed to the last phase (Figure 5m). In the last phase of the framework, we use the RNN network with three layers of GRU as a decoder (Figure 5p). Tokenizing and embedding layers convert all the preprocessed textual data to an integer vector before feeding the descriptions to the language model (Figure 5n,o). Finally, our model trains to describe all the textual and visual features of images by applying language modelling techniques (Figure 5q). The model steps in Figure 4 are summarized as follows:

1. Partitioning the image set into train, validation, and test subsets randomly
2. Applying image feature extractor models to extract visual features from the images (Figure 5a–j)
3. Extracting external knowledge for each image by searching the predicted labels from the previous step as a query in Wikipedia and adding it to the captions that already exist for the images in the dataset (Figure 5k–m)
4. Applying preprocessing methods to contextual data before feeding it to the RNN network, i.e., removing the punctuations numbers and wrapping each sentence around with “ssss” and “eeee” tokens to specify the beginning and end of sentences for the network (Figure 5n)
5. Transforming the textual features to the integers vector by tokenizing and embedding operations for training by the language model (Figure 5o)
6. Training language model for certain epochs based on its performance on validation data. During the training phase, the model predicts the next word of each word in the caption (Figure 5p,q)

After the training phase, the model is ready to evaluate test set images by extracting visual features and predicting the captions using a greedy search. Greedy search selects the word with the highest probability at each time step and uses it as the GRU input for the following time step until the end of the sentence is reached. In the next section, we will discuss the details of the experiments and the obtained results by the analyzed methods.

## 4. Experiments

This section reports the details of implementations and the results of the experiments conducted by different variations of models.

### 4.1. Dataset and Implementation Details

We use the MS-COCO dataset [15] to evaluate the proposed model in our experiments. MS-COCO contains 123,287 k images with five captions and 80 object categories for each image annotated by Amazon Mechanical Turk (AMT) workers. Since there are no available annotations for the test set, in this work, we used publicly available splits provided by Karpathy et al. [46]. We use 5000 images for validation and testing and the rest for the training set. All the models are implemented in Python version 3.6 and using the capabilities provided by Keras version 2.2.5 and TensorFlow version 1.15.0 deep learning libraries. Table 1 shows the parameters set for each network. The training was done using a machine equipped with two GeForce RTX 2080 GPU cards with 8 GB memory. The machine was installed with two GPUs, but for the experiments, only one was necessary.

### 4.2. Metrics

To compare our results to other baseline models, we measure the performance of the implemented models by the commonly used metrics, BLEU 1–4 [12], ROUGE [13], and METEOR [14].

**BLEU** is one of the popular metrics to evaluate the correspondence between generated sentences by humans and machines. This metric measures the maximum number of co-occurrence n-grams between reference and candidate sentences. Here, ‘n’ takes the value of 1, 2, 3, and 4 depending on the length of sentences. Each BLEU-N metric averages the calculated accuracies from n = 1 to n = N. It means that BLEU-1 is the accuracy of the description created for the image with the reference description based on 1-gram, BLEU-2 is the geometric mean of the calculated accuracies based on 1-gram and 2-gram, BLEU-3 is the geometric mean of the calculated accuracies based on 1-gram, 2-gram, and 3-gram, and so on.

**ROUGE** evaluates the performance of generated sentences by a machine based on their similarity to the reference sentences. This metric finds the longest subsequence of tokens between candidate and reference sentences and calculates how many tokens from the human reference summaries were duplicated in the machine-generated summaries. Unlike BLEU, which prioritizes precision, ROUGE is recall-oriented and can estimate correlated n-grams better than BLEU.

**METEOR** is the last evaluation metric in this paper. In this metric and the exact word match, the stemmed and wordnet synonym tokens are taken into account between the alignment of the candidate and the reference sentence.

**Baselines:** We provide two baseline approaches to verify the effectiveness of the models. The framework for the baseline is almost the same as the model in [25] as a baseline method, except that GRU replaces the LSTM language model. We used inception-V3 and VGG16 as the feature extractor method for the encoder part.

**Our approaches:** We assess different variations of our approach. CN + IncV3 utilizes the extracted features from the capsule network and inception-V3 as image features extractors. CN + VGG16 uses a VGG16 network rather than inception-V3 in the encoder. The Wikipedia knowledge base enriches the contextualized language model in this model. So, CN + IncV3 + EK and CN + VGG16 + EK are the models that use relevant external knowledge from Wikipedia. We also have performed additional experiments to check the importance of the capsule network in describing the content of images. To that end, we implemented IncV3 + EK and VGG16 + Ek methods to verify the effectiveness of the capsule network for image captioning models.

## 5. Results and Discussions

This section discusses the results from the different implementations of our framework and then compares them to state-of-the-art. Table 2 reports image captioning results for different implementations of our method on the MS-COCO dataset. The results demonstrate that the CN + IncV3 + EK model with capsule network and inception-V3 feature extractors can generate more human-like sentences by adding external knowledge to the language model. This model archives significantly better results in the overall metrics.

For the sake of brevity in explaining the results, we label BLEU 1, BLEU 2, BLEU 3, BLEU 4, ROUGE, and METEOR as B1, B2, B3, B4, R, and M, respectively. Specifically, the calculated metrics, B(1-4), R, and M for CN + IncV3 + EK method are 0.89, 0.74, 0.61, 0.54, 0.66, and 0.45, respectively. This result shows that the performance of this model is significantly better than the other implementations because it takes advantage of the capsule network and inception-V3 network as feature extractors and uses external knowledge to enrich the trainable contextual information for the language model.

When we implemented the model without external knowledge, we faced almost 13.5% performance degradation in B1. The degradation for other evaluation metrics is about 27%, 29.5%, 35.2%, 28.8%, and 22.2% for B (2-4), R and M, respectively, in the CN + IncV3 model.

The performance decreases about 33.7%, 40.5%, 39.3%, 46.3%, 53%, and 15.5% for all the B (1-4), R, and M, respectively, in the case we implemented VGG16 rather than inception-V3 in CN + VGG16 + EK model. Comparing the results between CN + IncV3 + EK as the best model and IncV3 + EK shows that including a capsule network improves the results. In this case, performance improvement is about 41.27%, 72.1%, 79.4%, 92%, 127.5%, and 45.2% for all the B (1-4), R and M metrics, respectively. Improving performance in these evaluation metrics when we implemented CN + VGG16 + EK and VGG16 + EK models is considerable. This improvement is as follows for B (1-4), R, and M, respectively: 55.3%, 63%, 68.2%, 61.1%, 34.8%, and 46.1%.

The results show that using VGG16 as a feature extractor is not as good as inception-V3 and decreases performance. Comparing CN + VGG16 and CN + VGG16 + EK models demonstrates adding external knowledge can enhance the performance of the language model. Comparing the evaluation metrics between these two models indicates 44%, 46.7%, 48%, 52.6%, 10.7%, and 11.8% improvement for B (1-4), R, and M, respectively.

**Figure 6:**
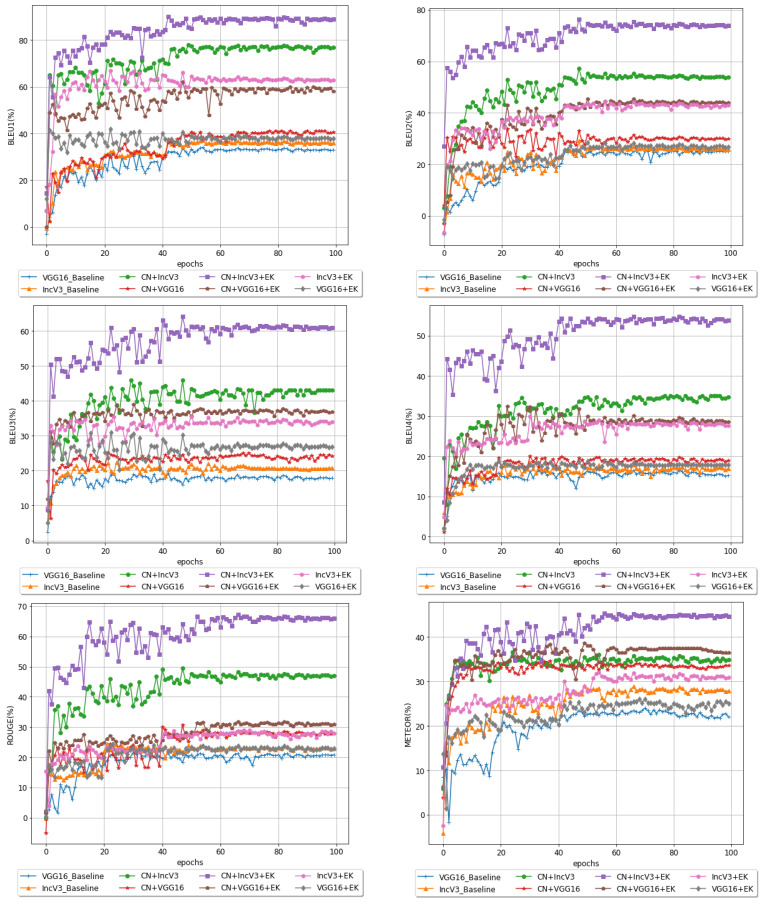
Comparative analysis on all the networks design using B1, B2, B3, B4, R, and M evaluation matrices.

A comparison between the different models from our experiments demonstrates the effectiveness of CN + IncV3 + EK as our best model. In Figure 6, all the introduced models on MS-COCO are compared with other baselines across BLEU 1, BLEU 2, BLEU 3, BLEU 4, ROUGE, and METEOR evaluation metrics. Comparing the results of applying all the models over the 100 training epochs shows that the performance of the model that includes external knowledge from Wikipedia and extracts image features by using inception-V3 and capsule network performs significantly better than the other models. According to the plots, it is evident that most of the models have converged after 60 epochs.

To prove the effectiveness of this model, we compare the result of the CN + IncV3 + EK method with state-of-the-art research. In Table 3, the bold numbers show that our best model outperforms previously published results on the MS-COCO “Karpathy” test split dataset.

Compared to our model, Ref. [47] has proposed an attention mechanism to leverage spatial features of an image to find salient objects. Tan et al. [48] proposed a tuning model with a small number of parameters in the RNN. Their model can produce a very sparse decoder for generating a caption preserving the performance of the method compared to their baseline. Zhang et al. [49] implemented a cooperative learning mechanism to combine two image caption and image retrieval modules while generating a caption. Then, during a multi-step refining process, they refined the image-level and object-level information to produce a meaningful caption.

Instead of using GRU as RNN, Yu et al. [50] proposed a model which employed a multimodal transformer as a language model in the decoder to generate a caption.

Contrary to our approach, Refs. [51,52] have focused on important image regions. Lu et al. [51] proposed an adaptive attention framework that could decide whether to rely on special attention to the image and when to attend to the textual image information.

In Ref. [52], Anderson et al. extracted a set of salient regions from the image by applying a bottom-up mechanism. They also implemented a top-down mechanism to determine the distribution of attention over the image to compute feature weightings in different regions.

Jiang et al. [53] proposed a framework that includes a recurrent fusion network. This fusion procedure is implemented between the encoder and decoder to exploit interactions among the represented features from the encoder part for creating a new set of vectors from decoder outputs.

### Qualitative Results

In this section, we present some examples to show the performance of the CN + IncV3 + EK method as our best model.

**Figure 7:**
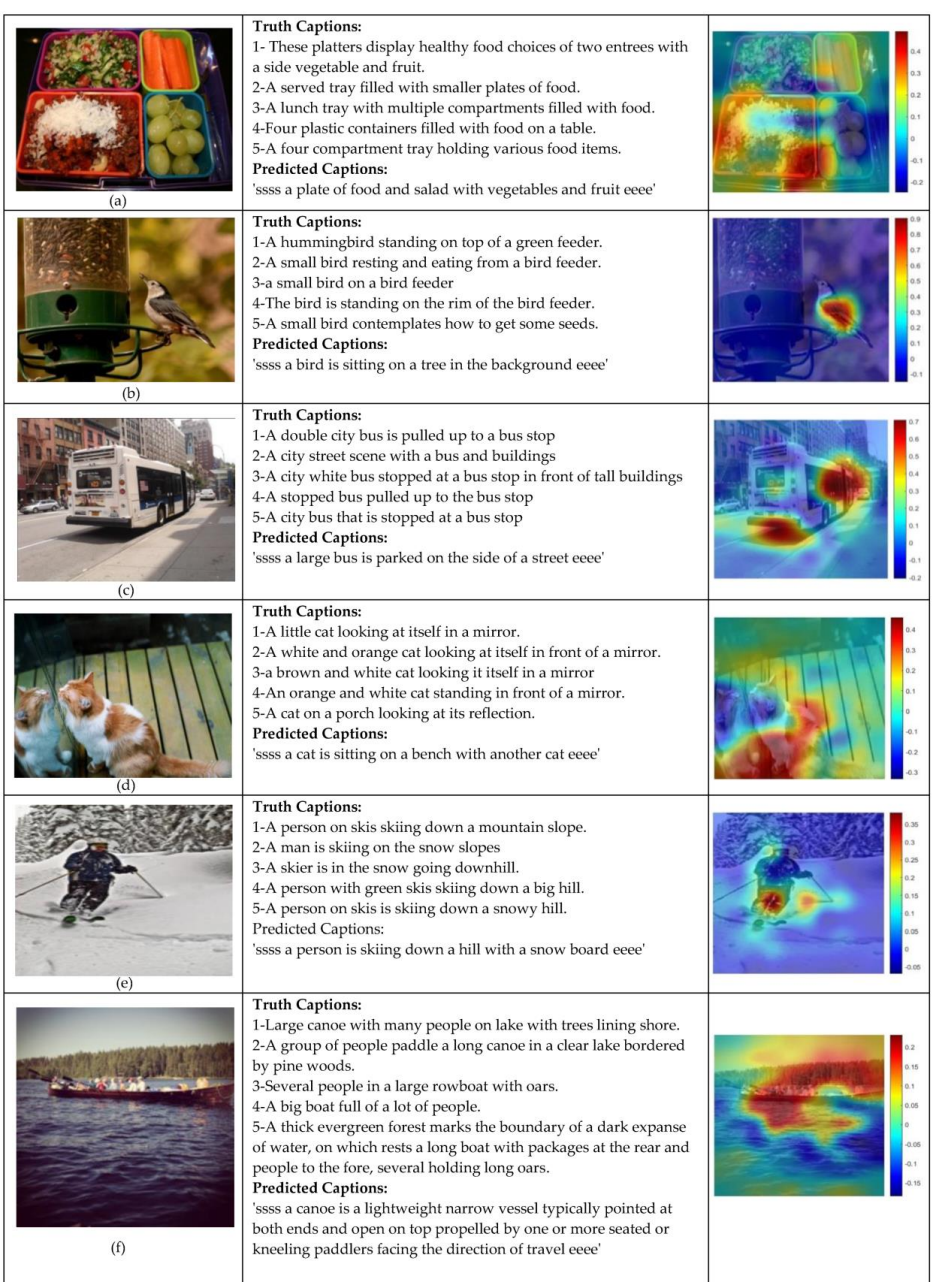
Generated examples by the best proposed model. (**a**) a plate of salad, (**b**) a bird on a bird feeder, (**c**) a bus at a bus station, (**d**) a cat in front of the mirror, (**e**) a person who is skiing, (**f**) a canoe on a lake.

We used the occlusion sensitivity function to visualize and localize the most important regions of the images for the network. The occlusion function computes sensitivity maps for CNNs. This function disturbs small input areas by replacing them with an occluding mask, typically a grey square, and moving the mask across the image to calculate the probability score of the given class. This method can highlight the most critical regions of the image for classification. Figure 7 shows some examples of occlusion sensitivity maps and the regions that provide more essential features for the network.

As demonstrated in Figure 7, using occlusion sensitivity helps us better understand features used by the network and provide insight into the reasons for the misclassified images. These examples show that CN + IncV3 + EK is the best descriptor model as it can generate more human-like sentences for each image.

We can appreciate the performance of our model on the generated caption for the photo in Figure 7a. The model identifies a good combination of all the objects within the image through the generated caption. In this example, there is a plate of ‘salad’ which is not mentioned in the five trained captions for the image, while the network has considered it in the predicted caption. We believe that it is the effect of using the Wikipedia database in the training phase to enrich the textual information of the network. Figure 7b shows that our network has identified bird feeder as a tree since they are almost similar. Moreover, the bird feeder concept was not in the trained descriptions by the network. Recognizing similar objects is one of the challenges of image captioning models. The occluded image also shows that our model focused on the bird region. In the Figure 7c photo, a bus is at a bus stop, and our model could detect it well. In this example, the model appropriately distinguished the position and status of attributes relative to each other.

Information about the posture and location of attributes is one of the advantages of using a capsule network in our model. An interesting point about Figure 7d photo is that our model has detected two cats in the image; however, the network did not notice one of them was the image of the first cat in the mirror. Moreover, the occluded image focused on the area of cats in the image. The photo of the person skiing (Figure 7e) has been described correctly, and the vital region of the image perfectly matches the generated caption in the occluded image. However, the ski board has been detected as a snowboard. Our model generates a longer and more detailed caption for Figure 7f. Using the Wikipedia database to enrich the description of attributes in the image is, to some extent, noticeable.

In summary, our proposed framework improves the performance of the image captioning process by employing a network that can produce more comprehensive features about relational information between all the objects in the image. Therefore, the model generates denser and more diverse captions. Moreover, we compensated for the low-resource language words by adding external knowledge from Wikipedia to the dataset. So, the decoder can benefit from rich-resource captions through the training process. In terms of the computation time, parallelizing the convolution layers in the enhanced version of the capsule network reduces the dimensions of the fed features fed to the primary capsules and accelerates the learning process.

## 6. Conclusions and Future Works

In this paper, we developed an encoder–decoder framework employing a novel parallelized capsule network as a feature extractor and the Wikipedia database as an external knowledge provider to establish if this approach can outperform state-of-the-art solutions. We implemented different architectures to produce contextual knowledge from images to achieve this. The models were trained on the MS-COCO dataset and evaluated based on BLEU (1–4), ROUGE, and METEOR scores. Our experimental setup has included two baseline models and is compared with several implementations to obtain a baseline performance. Our novel approach demonstrated that using a parallel capsule network as an encoder model provided a versatile image feature extractor.

We have demonstrated that the use of external knowledge further improved the results. Our best model was trained with the capsule network and inception-V3 as a feature extractor, with caption enrichment by an external contextual description. The results are the basis for future research that will generate more conceptual and specific descriptions by considering emotions in captions and using transformers in the decoder since this network have extraordinary performance in image captioning [54].

In the current framework, we have set hyperparameters either manually or by using previous settings studied in the literature. We leave it as future work to use hyperparameter optimization techniques, such as AutoML [55], to achieve optimal prediction performance. Another possible future direction that needs to be taken is to verify the robustness of the proposed method against noise through experiments. To this end, it may be useful to look at the effect of noise across different domains, for example, studied in [56]. We also intend to consider the diversity of the generated captions from various perspectives for assessing the performance of our models. An image may contain a variety of captions conveying different ideas and levels of detail, depending on the points of attention. As there is no standard methodology for evaluating captioning models, it is more appropriate to consider their diversity in order to assess their performance [57]. A multimodal learning approach or updating the training network with new datasets in different domains may be an interesting first step to incorporating diversity.

## Figures and Tables

**Table 1:** Specific parameters of the models in the evaluation.

Parameters	Networks		
	VGG-16	Inception-v3	CapsNet
Depth	16	48	8
Image size (pixel)	224 × 224	299 × 299	299 × 299
Solver (optimizer)	SGDM	RMSProb	ADAM
Loss function	cross-entropy	cross-entropy	MSE
Batch size	32	64	128
Learning rate	0.001	0.0001	0.001
Learning rate drop factor	0.1	0.1	0.5
Learning rate drop period	10	10	10
Momentum	0.9	0.9	0.9
Gradient threshold method	L2norm	L2norm	L2norm

SGDM: Stochastic gradient descent with momentum; Adam: Adaptive momentum estimation; RMSProb: root mean square propagation; MSE: mean squared error.

**Table 2:** The experimental results of implemented models. Bold text indicates the best overall performance.

Models	Metrics					
	BLEU1	BLEU2	BLEU3	BLEU4	ROUGE	METEOR
VGG 16 (Baseline)	0.33	0.24	0.18	0.16	0.21	0.24
IncV3 (Baseline)	0.36	0.26	0.21	0.17	0.23	0.28
CN + IncV3	0.77	0.54	0.43	0.35	0.47	0.35
CN + VGG 16	0.41	0.30	0.25	0.19	0.28	0.34
CN + IncV3 + EK	**0.89**	**0.74**	**0.61**	**0.54**	**0.66**	**0.45**
CN + VGG 16 + EK	0.59	0.44	0.37	0.29	0.31	0.38
IncV3 + EK	0.63	0.43	0.34	0.28	0.29	0.31
VGG 16 + EK	0.38	0.27	0.22	0.18	0.23	0.26

**Table 3:** Comparison of the best result to state-of-the-art.

Models	Metrics					
	BLEU1	BLEU2	BLEU3	BLEU4	ROUGE	METEOR
ours	**0.89**	**0.74**	**0.61**	**0.54**	**0.66**	**0.45**
Aneja et al., 2018 [47]	0.72	0.55	0.40	0.30	0.53	0.25
Tan et al., 2019 [48]	0.73	0.57	0.43	0.33	0.54	0.25
Wu et al., 2017 [25]	0.73	0.56	0.41	0.31	0.53	0.25
Zhang et al., 2021 [49]	0.75	0.62	0.48	0.36	-	0.27
Yu et al., 2019 [50]	0.81	0.67	0.52	0.40	0.59	0.29
Lu et al., 2017 [51]	0.75	0.58	0.44	0.33	0.55	0.26
Anderson et al., 2018 [52]	0.80	0.64	0.49	0.37	0.57	0.27
Jiang et al., 2018 [53]	0.80	0.65	0.50	0.38	0.58	0.28
Yan et al., 2020 [38]	0.73	0.53	0.39	0.28	0.56	0.25

## Data Availability

MS-COCO dataset can be found through https://cocodataset.org/.
